# Balloon laryngoplasty in children with acute subglottic stenosis: experience of a tertiary-care hospital

**DOI:** 10.1590/S1808-86942011000600006

**Published:** 2015-10-19

**Authors:** Claudia Schweiger, Mariana Magnus Smith, Gabriel Kuhl, Denise Manica, Paulo José Cauduro Marostica

**Affiliations:** 1 Otorhinolaryngologist; MSc in Medical Sciences; Pediatrician – Federal University of Rio Grande do Sul (UFRGS), Physician at the University Hospital of Porto Alegre (HCPA), Preceptor at the Medical Residency Program in Otorhinolaryngology HCPA; 2 Otorhinolaryngologist; MSc in Medical Sciences; Pediatrician - UFRGS, Preceptor at the Medical Residency Program in Otorhinolaryngology at the São Lucas Hospital of the Catholic University of Rio Grande do Sul; 3 Otorhinolaryngologist Adjunct Professor – Department of Otorhinolaryngology - UFRGS Preceptor at the Medical Residency Program in Otorhinolaryngology HCPA,; 4 Otorhinolaryngologist. MSc Student in Medical Sciences – Pediatrics - UFRGS, Fellowship in Laryngology and Voice - HCPA; 5 Pediatric Pneumologist. Post-doctorate in Pediatric Pneumology – Department of Pediatrics - UFRGS, Preceptor of the Medical Residency Program at HCPA, Professor – Department of Pediatrics - UFRGS, Preceptor of the Medical Residency in Pediatrics - HCPA

**Keywords:** balloon dilatation, child, laryngostenosis

## Abstract

**Abstract:**

Management of subglottic stenosis (SGS) in children is still a challenge to Otorhinolaryngologists. Balloon laryngoplasty (BLP) is an endoscopic procedure, first described in 1984 for the treatment of airway stenosis. It shows promising results and seems to be more effective than other procedures.

**Aim:**

To present our experience with BLP in children with SGS.

**Material and Method:**

Prospective study of children diagnosed with acute subglottic stenosis, i.e., stenosis with granulation tissue. They underwent direct laryngoscopy under general anesthesia and dilatation of the stenotic segment with angioplasty balloon. They were followed up and a second laryngoscopy was performed one week later.

**Results:**

Eight children were included in this study between June 2009 and October 2010. Four had Grade 3 SGS, three had Grade 2 SGS and one had Grade 1 SGS. By the second examination, two children presented with asymptomatic Grade 1 SGS, while the other six presented with normal airway and remained asymptomatic.

**Conclusion:**

BLP seems to be an effective treatment for acute SGS. We need more studies to refine our knowledge concerning efficacy rates, safety and indications for balloon dilatation.

## INTRODUCTION

Starting in the 1950's, prolonged endotracheal intubation (ETI) started to play an important role in the management of respiratory disorders in critically ill adults and children, and it became the main cause of laryngeal stenosis. Subglottic stenosis seems to be more common in children, because this is the narrowest region in the airway of people at this age range^1^.

The treatment of subglottic stenosis (SGS) in children continue to be a challenge for otorhinolaryngologists, and numerous open and endoscopic surgical techniques have been reported. Endoscopic techniques have the advantage of being less invasive and not leaving external scars; however, with variable success rates.

Among the endoscopic options for treatment, we found the CO2 or *yag laser* being used to resect the stenosis and dilatation using dilatation rods and stiff bronchoscopes and; more recently, angioplasty balloons.

The balloon laryngoplasty (BLP) is an endoscopic procedure, first described in 198^4^,^2^, used to treat stenosis of the upper airways. Such technique is being used, since then, to treat stenosis secondary to prolonged intubation, re-stenosis after laryngotracheal reconstructions and after cricotracheal resections with end-to-end anastomosis, with promising results^3^, ^4^, ^5^. A large variety of balloons have already being tested, among them we have the Fogarty embolectomy catheter and a number of angioplasty balloons^2^,^6^,^7^.

Dilatation may be carried out under direct visualization with laryngoscopy or bronchoscopy^7^ or by fluoroscopic control^3^,^5^. The procedure is palliative in some cases (turning a Myer & Cotton^8^ grade 3 SGS into a grade 2 or 1), and curative in others^9^.

Since its first description in the literature, BLP has been tested in subglottic stenosis, usually in small series of patients, but mostly showing promising results.

Our goal is to discuss our experience with BLP in pediatric patients with SGS after intubation and its evolution.

## PATIENTS AND METHODS

We included in the study eight pediatric patients diagnosed with post-intubation SGS grade 1-3 from Myer& Cotton^8^ in evolution, that is, SGS with granulation tissue, within the time frame between June of 2009 and October of 2010. These patients were found by means of a cohort study carried out by our study group, in which we did a nasopharyngolaryngoscopy in all the patients after intubation in our Pediatric ICU, since 2005. After nasopharyngolaryngoscopy, these patients were followed up and, if they had changes in the exam or upper airway obstruction symptoms in the follow up, they were submitted to direct laryngoscopy and, if any change was detected, they were submitted to specific treatment.

This study was approved by the Ethics Committee of our institution, under protocol number 05-266.

### Technique

**Figure 1:**
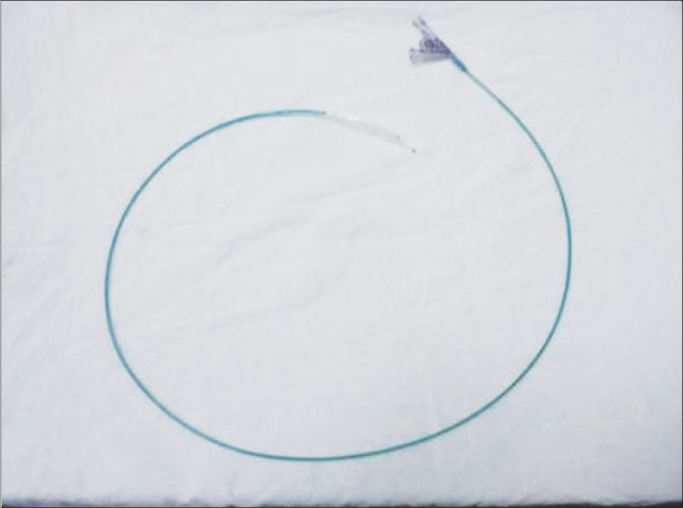
Angioplasty catheter used in the balloon laryngoplasty.

**Figure 2:**
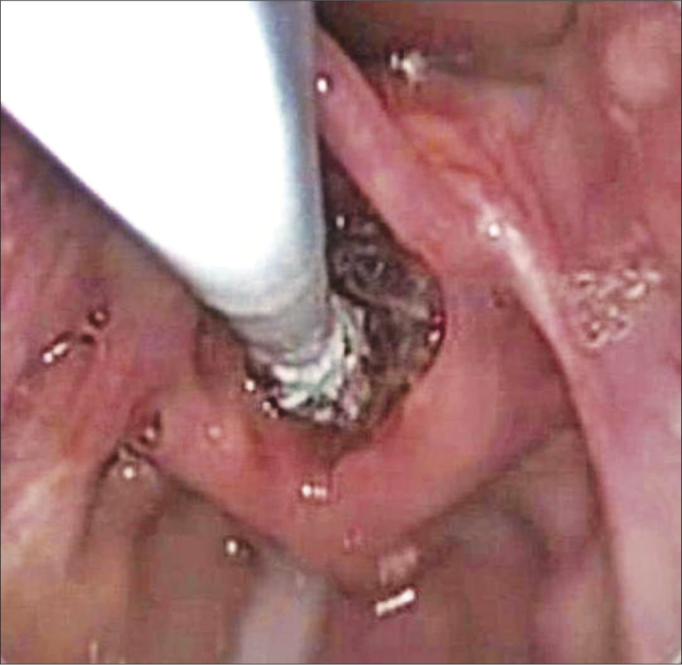
Direct laryngoscopy with a zero degree scope, showing the catheter being introduced in the larynx, with the balloon completely deflated

First, we carried out a direct laryngoscopy in order to diagnose and grade the SGS, using pediatric laryngoscopes and a scope of 0 degree and 4mm in diameter. The patient was sedated and remained in spontaneous ventilation during the procedure, receiving complementary oxygen through a nasal catheter. After the diagnosis and indication of balloon dilatation (acute stenosis in evolution, with granulation tissue, lower than grade 4), we introduced the balloon (angioplasty catheter - 4cm long and 10-14mm in diameter - Figure 1) through the laryngoscope, under direct view. The balloon was placed in the subglottis (Figure 2) and inflated with saline solution to a pressure of 2atm. The balloon remained inflated for 30 seconds and 2 minutes, and afterwards it was emptied and removed from the airway.

We then looked at the subglottis in order to see the immediate result. Should it be satisfactory, we removed the laryngoscope and the patient was awaken. Should the airway diameter be still not enough for a proper ventilation, the dilatation procedure was repeated. If after the second dilatation the airway was still not adequately dilated, the patient was referred to another type of treatment. The patient remained in oral steroids for 7 days after the procedure and had to take omeprazole for an indefinite period of time, until complete disease resolution. A new direct laryngoscopy was carried out to review the subglottis, 7 days after the dilatation procedure, and again whenever the patient presented symptoms. The patients were followed up for, at least, 6 months after the first dilatation.

## RESULTS

On Table 1, we can see the detailed clinical history from each one of our patients: age, cause of the endotracheal intubation, ETI time, symptoms presented, the time between extubation and BLP, the type and degree of stenosis, the type of balloon utilized and the final aspect of the patient's airway upon direct laryngoscopy.

**Table 1:** Clinical data of the patients and description of the BLP in each case.

Patient	Reason for the ETI	ETI	Symptoms	Time interval between extubation and the BLP	Type and grade of the stenosis	BLP	Final DL
LBS, female, 14 m	Pneumonia	7 days, without the balloon	Biphasic stridor	5 days	SGS Grade 2, with GT, concentric	12mm, 1m15s, 2 atm, 1X	Normal airway
KCA, female, 10 m	AVB	3 days, without the balloon	Biphasic stridor, weak cry, sternum retraction	6 days	SGS Grade 1, with GT, posterior	12mm, 2 min, 2 atm, 1X	Normal airway
RL, male, 9m	Meningococcemia	12 days, without the balloon	Biphasic stridor, sternum retraction	30 days	SGS Grade 3, with GT, posterior	10mm, 1 min, 2 atm, 2X	Normal airway
LAAS, male, 3m	AVB	14 days, without the balloon	Biphasic stridor, desaturation	Extubation failures	SGS Grade 3 + abundant glottic GT^a^	10mm, 2 min, 2 atm, 1X	Normal airway
GM, male, 2m	Pneumonia	5 days, without the balloon	Biphasic stridor, weak cry	60 days	SGS Grade 3, with GT, concentric	10mm, 2 min, 2 atm, 2X	Mature, Grade 1 SGS
NSS, female, 2 m	AVB	9 days, without the balloon	Biphasic stridor, sternum retraction	6 days	SGS Grade 2, with GT, posterior	10mm, 30s of duration, 1X	Normal airway
RLGS, male, 2m	AVB	3 days, without the balloon	Biphasic stridor, weak cry, sternum retraction	9 days	SGS Grade 2, with GT anterior and lateral to the left	10mm, 40 s of duration, 1X	Normal airway
WLC, male, 3m	AVB	8 days, without the balloon	Biphasic stridor, sternum retraction, weak cry, desaturation	14 days	SGS Grade 3, with GT, concentric	10mm, 30 s of duration, 2X	Mature, Grade 1 SGS

AVB: Acute Viral Bronchiolitis. ETI: endotracheal intubation. DL: direct laryngoscopy. BLP: size of the dilatation balloon (external diameter of the balloon), dilatation duration (balloon inflated, completely obstructing the patient's airway), balloon pressure, number of dilatations performed. GT: granulation tissue SGS: subglottic stenosis. ^a^: This patient required a tracheostomy because the glottic granulation tissue (GT) remained obstructive for 2 weeks after subglottic dilatation. After this period, the GT resolved completely and the patient was decannulated. The subglottic stenosis resolved completely after BLP.

Our complete resolution rate for the stenosis in evolution was of 75% (six in eight patients), but all the children were asymptomatic during follow up, even those with Grade 1 residual stenosis, not requiring additional treatment up to this moment.

**Figure 3:**
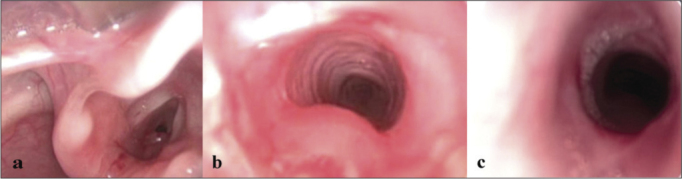
Patient with grade 3 subglottic stenosis and granulation tissue (3A), submitted to balloon laryngoplasty (3B – image taken immediately after dilatation, and 3C – image taken 2 weeks after the procedure, with residual grade 1 stenosis - posterior).

On Figure 3, we can see the aspect of the direct laryngoscopy from patient WLC before BLP (Grade 3 SGS), immediately after the dilatation, and two weeks afterwards (asymptomatic Grade 1 SGS).

We had no severe complications during the procedures.

## DISCUSSION

We presented here our series of eight children with acute stenosis after intubation, submitted to dilatation with an angioplasty balloon. Most of our patients were infants, previously healthy, with a short ETI time (3-14 days) in whom endotracheal tubes without a circumferential balloon was utilized. One of them had extubation failure and, for that, was submitted to direct laryngoscopy, and all other started having upper airway obstruction symptoms between 5 and 60 days after the extubation, being also submitted to diagnostic laryngoscopy.

Endoscopic techniques used to treat stenosis are once again being stressed. Balloon laryngoplasty seems to be more effective than the other means of dilatation, because the entire force employed is radial, towards the stenosis area. The lack of shearing forces reduces subglottic trauma, both at the mucosa and in the deeper areas, thus reducing the changes of re-stenosis^10^. Moreover, because of the small deflated balloon diameter, it can be passed through extremely narrow areas, without causing trauma^9^. Just as in dilatations with rods and rigid bronchoscopes, balloon dilatation is also more successful when one is dealing with immature scar tissue (granulation tissue), although there are reports of its efficiency in subglottic stenosis^4^,^6^. Dilatation my require to be repeated a few times in order to reach the desired outcome^2^, ^3^, ^4^,^7^.

The first case of BLP for SGS described in the literature was in 1985, when Axon et al. Reported a case of a symptomatic four-year old diagnosed with grade 2 SGS after intubation^10^. They used a balloon with 6mm in diameter and 15mm long, inflated at 6atm of pressure, leaving it in the subglottis until the child's hemoglobin saturation dropped to 80%. The child remained asymptomatic after 15 months of observation.

In 2007, Durden& Sobol published a series of 10 pediatric patients with SGS submitted to balloon dilatation^9^. About half of the cases were patients with acute SGS, still bearing granulation tissue. They showed that BLP is an effective technique to acutely secure a severely compromised airway and it seemed to completely resolve symptoms in a large number of children with SGS, with success indices comparable to those of laryngeal reconstruction (about 70%). The authors did not have complications in their dilatation procedures and reported that these do not rule out the possibility of doing a laryngotracheal reconstruction later one, should that be the case. They also state that BLP is not effective for long-standing SGS, congenital cases or those involving cartilage. Nonetheless, half of their favorable outcome cases were associated with mature SGS without granulation tissue. In our study we did not include patients with mature stenosis without granulation tissue.

Despite the small sample, our data also showed that BLP is effective to treat evolutional SGS (with granulation tissue) in children. We showed a high rate of total resolution for the subglottic lesion in six of the eight patients (75%). Before we started to use BLP in our clinic, these patients with respiratory distress caused by post-intubation subglottic stenosis were referred to direct laryngoscopy and, usually, to tracheostomy at the same time. Some were eventually cured, but others developed a mature stenosis and required later laryngotracheal reconstructive surgery. With BLP we were able to avoid tracheostomy in seven of the eight patients, and we avoided laryngotracheal reconstruction in all of them.

Since we used oral steroids and omeprazole in all the patients, we could not infer on which effects were associated to this medication and which were associated with BLP alone. Nonetheless, when we treated granulation tissue patients with omeprazole and steroids only, our rate of tracheostomies was higher. Thus, the benefit of the BLP is, at least, airway stabilization and tracheostomy avoidance until medication is able to reduce the formation of granulation tissue.

Only two children remained with residual, nonobstructive SGS. These children remained in follow up at our clinic and are asymptomatic until current days.

One of the children required tracheostomy after BLP, but this was due to glottic obstruction by abundant granulation tissue, and not to subglottic obstruction, which was completely resolved with the dilatation procedure.

We had no complications during the procedures, despite the fact that these have been reported, such as atelectasis, bleeding and cricoid rupture, in studies with animals^11^.

## CONCLUSION

BLP seems to be the safest and most efficient procedure to treat post-intubation SGS in children. Further studies are required in order to know what is the real success rate of such procedure, which patients benefit from the technique, the proper balloon diameter for each age range, its ideal resting time in the subglottis and the maximum inflation pressure one can use.
